# Development and Validation of a Workplace Age-Friendliness Measure

**DOI:** 10.1093/geroni/igaa024

**Published:** 2020-07-01

**Authors:** Raphael Eppler-Hattab, Israel Doron, Ilan Meshoulam

**Affiliations:** 1 Department of Gerontology, Faculty of Social Welfare and Health Sciences, University of Haifa, Israel; 2 Faculty of Management, University of Haifa, Israel

**Keywords:** Aging workforce, Older workers, Organizational culture, Scale development

## Abstract

**Background and Objectives:**

Measuring the extent to which the culture of organizations can be considered age-friendly is a significant anchor in the constructive inclusion process of older workers in workplaces, given the consistent aging of the workforce. Hence, the purpose of this research was to develop a novel, comprehensive, and theoretically driven measure of workplace age-friendliness.

**Research Design and Methods:**

Three multiphased, multisourced studies were conducted: a qualitative assessment procedure and 2 separate quantitative field surveys of individual-level perceptions.

**Results:**

A 24-item scale of workplace age-friendliness was developed, consisting of 4 dimensions that represent the different ways in which organizational culture aligns with an aging and older workforce: age-friendly core culture, development, wellness, and flexibility. Confirmatory factor analysis verified that a 4-factor structure is the most appropriate solution, with all dimensions having acceptable internal consistency. Preliminary evidence of construct validity is also presented.

**Discussion and Implications:**

The measure developed in this study may serve researchers as well as practitioners in the field of aging and work. Further implications and limitations of using this instrument in future empirical study on workplace age-friendliness are discussed.

**Translational Significance:** The Workplace Age-Friendliness Measure may be used to advance and accommodate the constructive and healthy inclusion of aging and older workers in workplaces. It can be adopted as part of the involvement of policymakers at the organizational and/or national level charged with promoting equitable employment for older persons.

Workplace age-friendliness refers to the extent to which an organization maintains the employability of its older workers by embracing an organizational culture in which they are accepted and treated according to their competencies and needs (Eppler-Hattab et al., 2020). The need to measure the extent to which workplaces are age-friendly becomes increasingly important against the backdrop of the aging of the workforce and prolonged working life. In particular, recent evidence suggests that maintaining age-inclusive workplace environments can have positive implications for organizational productivity, performance, and innovation (Backes-Gellner & Veen, 2013; Boehm et al., 2014). These trends are not surprising given the increase in the share of older workers (55 plus) in the labor market, which in certain developed countries is expected to reach a potential of 32%–40% by 2030 (United Nations [UN], 2017).

Although an age-friendly culture can, in principle, be applied to workers of any age, the construct of age-friendliness in the workplace is contextualized as one aimed primarily at older workers (Appannah & Biggs, 2015; Eppler-Hattab et al., 2020; Wöhrmann et al., 2018). There are two main reasons for this perspective. First, the literature provides evidence of a considerable underutilization of older workers, and the adverse consequences of work-related age stereotypes that affect older workers significantly more than workers in other age groups (Harris et al., 2017). As a result, while recognizing that younger age groups may encounter their own age disadvantages at work (Taylor et al., 2016; Twenge, 2010), and without eliminating their needs, the meaning given to workplace age-friendliness is providing support for aging as part of the constructive inclusion of older workers in organizations. Second, reinforcing an age-friendly culture can convey a positive message to workers of different generations, thereby fostering continuous employment capacity across the working life span, and enhancing intergenerational relations and collaborations in the workplace (Burke, 2015).

In view of these consequences, a number of constructs have been presented in the literature pertaining to assessing workplace suitability for older workers. However, the existing constructs have three major limitations: relying on common perceptions and expectations of older workers that are not specifically related to a particular organization (Armstrong-Stassen, 2008), using a list of practices that are not necessarily culture-driven (Kooij et al., 2014), or adopting a generic age approach that is not uniquely adapted to aging and older workers (Boehm et al., 2014; Marchiondo et al., 2016; Pitt-Catsouphes et al., 2015; Zacher & Yang, 2016). Yet, we acknowledge that other frameworks for assessing workplace adjustments to an older workforce are evolving against the complexity of working longer in an era of longevity, such as the Later-Life Work Index (Wilckens et al., 2019; Wöhrmann et al., 2018). In this study, we present the development of a comprehensive operational scale for quantitatively measuring workplace age-friendliness from an organizational culture perspective.

## Theoretical Background

The concept of age-friendliness in the workplace is drawn from the theoretical grounds of organizational culture and climate. As a starting point, it is assumed that an organizational culture that is age-friendly is based on the fundamental assumptions and deeply rooted values that guide the members of the organization on how to think and act with respect to the integration of older workers. This interpretation is derived from the accepted definition of organizational culture outlined by Schein (2004), according to which the espoused values shared among the organization’s members are a central component of a distinct and specified culture.

Furthermore, as Ostroff et al. (2013) elaborate, organizational culture can motivate the emergence of supportive policies, practices, and procedures (hereinafter: practices) that demonstrate the ways in which culture is manifested. In this process, the perceived work environment shared among the organization’s members with regard to these supportive practices is conceptualized as an organizational climate (Schneider et al., 2013). This means that if supportive practices are embedded in the organization, they serve as the linking mechanism between culture and climate, and thus an alignment may be maintained between culture, practices, and climate. For example, an age-friendly culture may be the basis for cultivating age-friendly practices which, in turn, create shared perceptions that emerge into an age-friendly organizational climate.

**Figure 1.**
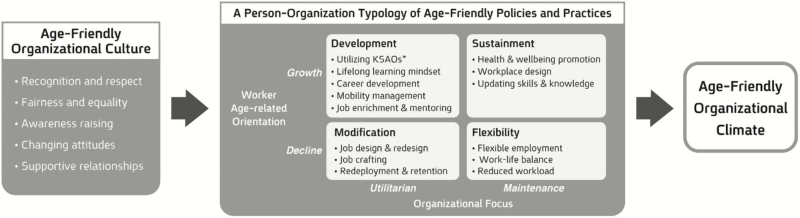
A multidimensional model of the Age-Friendly Workplace (reprinted from Eppler-Hattab et al., 2020). *Knowledge, skills, abilities, and other interests and values.

According to the theoretical model of the Age-Friendly Workplace (AFW; Figure 1), this study develops a measure of the two edges of this relationship, namely, the perception of espoused values that are age-friendly on the one hand and the perception of age-friendly practices that nurture an age-friendly climate on the other hand. As a cultural facet, the core values of an AFW include principles such as recognition and respect of older workers, fairness and equal opportunities in human resource processes, and values reflecting supportive relationships. In the implied workplace environment facet, four clusters of organizational practices may generate an age-friendly climate: development, sustainment, modification, and flexibility.

Each of these clusters contains a bundle of supportive practices of functional significance. Development practices include lifelong learning processes, in which the changing Knowledge, Skills, Abilities, and Other interests and values (KSAOs) of older workers are utilized. Sustainment practices involve the protection and promotion of health and well-being in the workplace, accompanied by training and qualification processes to update and upgrade professional skills and knowledge. Modification practices are about ways to improve performance and efficacy, mainly through job design and redesign. Finally, flexibility practices provide flexible employment arrangements for older workers who prefer or need to change their work–life balance.

The above clusters are adapted to a range of individual and organizational conditions in which an age-friendly organization engages with older workers. Development and sustainment practices are designed for older workers who continue to develop and grow, whereas modification and flexibility practices are aimed at older workers who reduce their work involvement (based on life-span development theory; Heckhausen et al., 2010). Development and modification practices allow the organization to directly benefit from utilizing the KSAOs of older workers, whereas sustainment and flexibility practices require the organization to maintain the existing KSAOs of older workers (as derived from the Resource-Based View framework; Wright et al., 2001). Given this framework, we propose that workplace age-friendliness can be captured with five interrelated factors: core culture, development, sustainment, modification, and flexibility.

## Present Study

The purpose of the present study is to produce a Workplace Age-Friendliness Measure (WAFM) based on a multidimensional model of the AFW (Eppler-Hattab et al., 2020). This measure may serve both researchers and policymakers as an instrument for investigating the constructive inclusion of an aging and older workforce in organizations. In order to develop a reliable and valid measure of workplace age-friendliness, three multiphased studies were conducted, conforming to the accepted scale development literature (DeVellis, 2016; Hinkin, 2005). In the first study, we generated a broad pool of items based on the AFW model. The items were reviewed and assessed by a panel of experts and revised accordingly. In the second study, a subset of 31 items of workplace age-friendliness was further refined to 24 items by analyzing the interitem and criterion-based correlations, and variances of each item, and examining the factor structure of all items using exploratory factor analysis. A third study was subsequently conducted using confirmatory factor analysis (CFA) to verify the proposed dimensionality of the remaining 24 items. Finally, convergent and discriminant validity indices were produced and analyzed for the subscales. These studies are described in the following sections.

## Procedures and Results

### Study 1: Instrument Development

#### Phase 1: Item generation

The purpose of this phase of the study was to create an inclusive pool of statements and examples of the ways in which an organization maintains the employability of older workers, so that together they reflect the manifestation of an age-friendly culture. In line with this purpose, we relied on two sources to obtain content validity. The first source was members of the “Late Adulthood” steering committee of the Israeli Society for Human Resources Management (ISHRM). The second source was previous theoretical and empirical literature that provides good qualitative analysis of age-friendly practices (Ciampa & Chernesky, 2013; Kooij et al., 2014; Loretto & White, 2006; Midtsundstad & Bogen, 2014). An initial pool of 48 items of human resource practices and supportive culture statements was obtained, taking into account considerable redundancy.

##### *Procedure 1*.

—We documented and summarized related materials from the “Late Adulthood” committee meetings, attended by two key authors of this study. The members of the committee were 15 highly experienced human resource directors and experts. The members had an average age of 59 years (*SD* = 8.92). Sixty-two percent of the members were women. The committee’s meetings were held for a period of 2 years and consisted of two main stages. First, a targeted exploratory survey was conducted among human resource managers from 20 large organizations in the business and public sectors (e.g., high-tech, secondary industry, higher education, and banking) dealing with organizational practices for preretirement workers. The survey included some open-ended questions to understand the ways in which organizations engage with employees older than 55 years to leverage their capabilities in terms of best practices embedded. Second, based on a preliminary picture provided and participants’ knowledge, an outline was developed for an organizational toolkit of principles and best practices for older workforce management. A total of 20 best practices were obtained from this process, including, for example, age-based diversity in the workplace, professional retraining, employment flexibility, learning and regeneration strategies, intergenerational mentoring, raising awareness in age-based recruitment, and recognition programs.

##### *Procedure 2*.

—Independently of Procedure 1, we consulted existing scholarly literature on organizational adjustments for older workers to produce a separate set of items, using a deductive item-generation approach appropriate to a domain with sufficient theoretical grounds (Haynes et al., 1995; Hinkin, 2005). Using our definition of workplace age-friendliness as a reference point, we generated items on the basis of four considerations.

First, we consulted the literature on the needs and expectations of older workers from their present or future workplace, as measured in previous studies (Armstrong-Stassen, 2008; Kooij et al., 2014). For example, two of the most prominent issues older workers seek are recognition and respect (in the context of not being transparent), and flexibility in terms of the scope of the position and the number of hours worked (Pitt-Catsouphes et al., 2009).

Second, we were inspired by items measured on recurring themes of positive age-related organizational practices from existing literature (Armstrong-Stassen, 2008; Boehm et al., 2014; Kooij et al., 2010). For example, the age-inclusive human resource practices scale developed by Boehm et al. (2014) contains an item reflecting the extent to which an organization offers “equal opportunities to be promoted, transferred, and to make further career steps irrespective of one’s age” (p. 702), which captures a certain indication of an age-friendly climate.

Third, we have assembled a mixture of items that are indicative of exclusively effective practices for older workers, and those that could effectively be applied to all workers, but are increasingly important to workers as they age. Finally, we formulated the items so that employees could identify them as being related to an age-friendly organizational culture. A total of 48 items were obtained from this process. The items were then reviewed for redundancy with the 20 best practices obtained in Procedure 1, after which a combined set of 48 items remained. At this point, items were classified according to the five-dimensional model of the AFW, of which 15 were classified into core culture, seven for development, 11 for sustainment, seven for modification, and eight for flexibility.

#### Phase 2: Item review

##### Sample.

—The 48 items were reviewed by 17 judges with different but related areas of expertise: nine were human resource directors in large organizations, five were senior organizational consultants, and three were managers or CEOs of organizations providing human resource services. The average age of the judges was 57 years (*SD* = 6.17), and their average professional seniority was 27 years (*SD* = 3.94). Seventy-six percent of the judges were women. Thirteen of the judges had a master’s degree in organizational behavior sciences or business administration, and three had a doctoral degree in behavioral and management sciences.

##### Procedure 1.

—The judges reviewed the items based on several criteria. First, the judges rated each item as to whether it represents our definition of workplace age-friendliness. This process was accompanied by a brief theoretical background and some methodological emphases to ensure face validity. The judges rated each item on a three-point scale, ranging from a rating of 1 for “does not represent the definition” to a rating of 3 for “clearly represents the definition” (Hardesty & Bearden, 2004). Second, the items were reviewed by the judges in terms of their literal clarity and reasonableness, to be revised accordingly, if necessary. Finally, the judges made general comments concerning the totality of the construct based on their overall professional experience and their familiarity with Israeli and/or global organizations.

##### Procedure 2.

—The judges’ ratings and review were examined using a number of indices to determine the items to be retained, removed, or reedited. Initially, Kendall’s coefficient of concordance (Legendre, 2010) was used to examine the degree of agreement between the judges regarding the representation of the theoretical concept by all items. A relatively low coefficient was obtained, indicating that the null hypothesis (a consensus among all judges’ ratings) was rejected (Kendall’s *W* = 0.192; χ ^2^ = 126.46; *df* = 47; *p* < .00). However, this result involved high agreement among judges on several items and lower agreement on others. The high-agreement items were mainly those that are exclusively related to older workers (e.g., retirement planning, job redesign, mentoring; hereinafter: exclusive items), whereas the low-agreement items were those that are not exclusively related to older workers (e.g., employment flexibility, career development, health and well-being promotion; hereinafter: unexclusive items; Froidevaux et al., 2020).

Therefore, we then considered the inclusion of each item separately, based on three indices: the average and standard deviation of the ratings of each item, and the percentage of judges stating that each item clearly represents the definition of age-friendliness (Hardesty & Bearden, 2004). For exclusive items, we required stricter agreement among judges than for unexclusive items. Accordingly, for exclusive items, ratings average of at least 2.70, standard deviation of less than 0.60, and a percentage of judges stating a clear representation of at least 80% were taken for inclusion, whereas for unexclusive items, ratings average of at least 2.50, standard deviation of less than 0.75, and a percentage of judges stating a clear representation of at least 65% were taken for inclusion. Reedited items were treated similarly, given content consciousness. As a result of this process, 31 items survived according to the following breakdown: 12 in the core culture dimension, five in development, five in sustainment, four in modification, and five in flexibility.

### Study 2: Instrument Refinement

#### Sample and procedure

The sample included the evaluations of human resource managers and professionals from 122 medium and large organizations in Israel, from diverse industries (e.g., hotelkeeping, health services, high-tech, consumer goods, and transportation). Forty-nine percent of the respondents were ISHRM members who represent larger organizations with greater awareness of the issue. Seventy-eight percent of the respondents were women. Nineteen percent of the respondents were between the age of 30 and 40, 32% between 40 and 50, 34% between 50 and 60, and 15% older than 60.

To facilitate the evaluation process, an online survey was conducted, composed of a list of 32 statements of workplace age-friendliness. The survey consisted of the remaining 31 items from Study 1, plus an intuitive statement reflecting an overall conceptual assessment of workplace age-friendliness to be used as an additional criterion for inclusion of items (“Considering all aspects known to me, it can be said that the organizational culture of my organization is friendly to the employment of older workers”; hereinafter: AFW item). Participation in the survey was voluntary, while ensuring personal and organizational anonymity.

The participants were asked to indicate on a seven-point Likert scale ranging from “strongly agree” to “absolutely disagree” the extent to which each of the statements is consistent with their organization’s values and conduct in addressing older workers. The participants were told that for the purpose of the study, an older worker is an employee aged 55 and older (in line with McCarthy et al., 2014).

#### Phase 1: Item selection process

Evaluation and selection of items were made on the basis of three criteria: item-total correlations, item variances, and concurrent criterion-based correlations. First, items with average to high interitem correlations (mostly between .30 and .60) with items of the same theorized dimension were selected to be included in the subscales, as poorly correlated items might not measure the same construct, whereas overly correlated items indicate unnecessary repetition (DeVellis, 2016). Second, the correlations between the AFW item and all other items were also analyzed for item inclusion, as cross-validation between explicit and implicit psychological constructs may help in reinforcing the validity of the underlying construct (Clark & Watson, 1995). Third, the variances of items were inspected in the selection process so as not to be too low, thus producing sufficient distinction between respondents on the age-friendliness scale.

##### Results.

—With the exception of two items, the correlations between the AFW item and the other items were above a required threshold of .20 and significant at a level lower than .01. The two low-correlated items with the AFW item, eliminated accordingly, represent assistance for the transition of older workers to retirement, implying that support for retirement is not a necessary component of workplace age-friendliness. The variance of all items ranged between 1.80 and 4.35, a reasonable product of the variety of organizations represented in the sample. The tripartite selection process resulted with the removal of six items, thus 25 items remained. Table 1 displays the means and standard deviations of these items, as well as their Kendall’s tau-b correlations (Gibbons, 1993) with the AFW item.

**Table 1.** Means, Standard Deviations, and Zero-Order Correlations With the AFW Item in Study 2

	Item	*M*	*SD*	τ _b_
1.	My organization treats older workers fairly and equally.	5.91	1.34	.66
2.	In my organization, there is no age discrimination in processes such as recruitment, promotion, and dismissal.	5.55	1.59	.65
3.	Managers in my organization are a personal example of the wish to recruit and retain workers of all ages, including older workers.	5.13	1.73	.66
4.	In my organization, there is a positive atmosphere toward the employment of older workers.	5.58	1.44	.76
5.	My organization promotes multiage diversity in the organizational workforce.	5.18	1.72	.64
6.	My organization makes sure that older workers are recognized and respected no less than other workers.	5.87	1.43	.55
7.	My organization shows responsibility for older workers who have long contributed to the organization.	5.72	1.45	.56
8.	Older workers in my organization are not the first priority for dismissal during organizational change or downsizing.	5.50	1.73	.57
9.	Older workers in my organization are not pressured to vacate their place and retire early.	5.58	1.53	.38
10.	My organization allows older workers to update and upgrade their knowledge and skills as part of their job.	5.57	1.48	.51
11.	In my organization, older workers are encouraged to acquire more new skills appropriate for changes in their professional field.	4.70	1.59	.23
12.	In my organization older workers are encouraged to serve as mentors for other employees.	5.20	1.47	.53
13.	My organization allows older workers to continue to develop throughout their working lives.	5.23	1.52	.48
14.	In my organization, older workers are encouraged to initiate changes in their jobs, in line with the needs of the organization.	4.53	1.64	.24
15.	My organization knows how to benefit from the total knowledge, skills, and abilities of older workers.	5.61	1.45	.49
16.	My organization allows older workers to make changes in their professional work.	4.61	1.60	.31
17.	My organization takes care and acts to promote the health and well-being of older workers.	4.57	1.55	.45
18.	My organization encourages older workers to participate in health promotion activities.	3.78	1.60	.49
19.	My organization works to raise awareness and change attitudes toward continuing work at older ages.	4.53	1.79	.67
20.	In my organization, older workers are offered job changes, if necessary, to better fit their abilities.	4.38	1.72	.35
21.	My organization organizes the work so that older workers remain in the organization in optimal functioning.	4.73	1.60	.38
22.	When required, my organization helps to reduce or adapt physical or psychological efforts to the abilities and needs of older workers.	4.46	1.69	.50
23.	In my workplace, older workers are given flexibility in choosing the range of hours worked.	4.18	1.94	.27
24.	In my workplace, older workers are given flexibility in choosing the scope of the position.	4.38	1.91	.51
25.	In my workplace, older workers are given flexibility in choosing the job location.	3.40	1.74	.29

*Notes:* AFW = Age-Friendly Workplace. Responses ranged from 1 (*absolutely disagree*) to 7 (*strongly agree*). All Kendall’s tau-b (τ _b_) correlations are significant at *p* < .01 (two-tailed). *N* = 122.

#### Phase 2: Exploratory factor analysis

We performed a principal factor analysis on the remaining 25 items, in order to analyze the interrelationships of the items and investigate the underlying factor structure, while examining additional items to be removed (Bryant & Yarnold, 1995; Fabrigar et al., 1999). In this procedure, the responses of ISHRM members were given double weight (using SPSS weight cases procedure; DeVellis, 2016; Kalton & Flores-Cervantes, 2003) that matched the estimated size of organizations represented, due to their deeper understanding of the issue in light of their familiarity with the “Late Adulthood” committee and its work. Given our proposed five-dimensional workplace age-friendliness model, we used the principal axis factoring method, appropriate for latent theorized constructs (Fabrigar & Wegener, 2012). The rotation method used was Varimax with Kaiser Normalization and rotation converged in six iterations. To ensure that each item is uniquely represented in the construct of each latent factor, we used a factor loading of .50 as the minimum cutoff, while maintaining a difference of at least .10 between loadings for any given item across factors (Henson & Roberts, 2006).

##### Results.

—As given in Table 2, one item (Item 16) did not meet the latter criteria and thus was removed, leaving us with 24 items. This item has a .50 borderline loading on the development factor and is not uniquely interpreted across development and flexibility factors. The resulting factor structure contains four factors for further validation, labeled core culture (nine items), development (six items), wellness (six items), and flexibility (three items). The total variance explained by these factors is 66.3%.

**Table 2.** Principal Axis Factor Analysis in Study 2

	Item	1	2	3	4
1.	My organization treats older workers fairly and equally.	**.86**	.20	.14	.11
2.	In my organization, there is no age discrimination in processes such as recruitment, promotion, and dismissal.	**.79**	.23	.07	.23
3.	Managers in my organization are a personal example of the wish to recruit and retain workers of all ages, including older workers.	**.77**	.16	.18	.33
4.	In my organization, there is a positive atmosphere toward the employment of older workers.	**.75**	.20	.26	.15
5.	My organization promotes multiage diversity in the organizational workforce.	**.71**	.12	.25	.14
6.	My organization makes sure that older workers are recognized and respected no less than other workers.	**.70**	.37	.19	.15
7.	My organization shows responsibility for older workers who have long contributed to the organization.	**.68**	.37	.29	.22
8.	Older workers in my organization are not the first priority for dismissal during organizational change or downsizing.	**.68**	.38	.09	.22
9.	Older workers in my organization are not pressured to vacate their place and retire early.	**.63**	.38	.18	−.07
10.	My organization allows older workers to update and upgrade their knowledge and skills as part of their job.	.46	**.66**	.21	.12
11.	In my organization, older workers are encouraged to acquire more new skills appropriate for changes in their professional field.	.43	**.64**	.26	.23
12.	In my organization, older workers are encouraged to serve as mentors for other employees.	.42	**.61**	.13	.27
13.	My organization allows older workers to continue to develop throughout their working lives.	.37	**.60**	.31	.38
14.	In my organization, older workers are encouraged to initiate changes in their jobs, in line with the needs of the organization.	.24	**.60**	.21	.42
15.	My organization knows how to benefit from the total knowledge, skills, and abilities of older workers.	.43	**.54**	.14	.27
16.	My organization allows older workers to make changes in their professional work.	.18	.50	.40	.43
17.	My organization takes care and acts to promote the health and well-being of older workers.	.18	.15	**.80**	−.04
18.	My organization encourages older workers to participate in health promotion activities.	.11	.12	**.76**	.16
19.	My organization works to raise awareness and change attitudes towards continuing work at older ages.	.32	.10	**.62**	.42
20.	In my organization, older workers are offered job changes, if necessary, to better fit their abilities.	.25	.37	**.55**	.41
21.	My organization organizes the work so that older workers remain in the organization in optimal functioning.	.41	.34	**.53**	.30
22.	When required, my organization helps to reduce or adapt physical or psychological efforts to the abilities and needs of older workers.	.22	.27	**.52**	.41
23.	In my workplace, older workers are given flexibility in choosing the range of hours worked.	.18	.21	.09	**.82**
24.	In my workplace, older workers are given flexibility in choosing the scope of the position.	.13	.16	.29	**.71**
25.	In my workplace, older workers are given flexibility in choosing the job location.	.15	.19	.08	**.64**
	Eigenvalue	12.73	2.41	1.62	1.14
	% variance explained (rotated factors)	25.30	14.62	13.36	12.98

*Notes:* Numbers in boldface indicate dominant factor loadings. 1—Core culture, 2—Development, 3—Wellness, 4—Flexibility. *N* = 182.

### Study 3: Instrument Validation

#### Sample

Two samples were used for the validation process. The main sample was composed of 448 employees from five organizations operating in different Israeli industries: chemicals, media, agriculture, nursing services, and finance. This sample provided self-perceptions of the remaining 24 items of workplace age-friendliness from Study 2, as well as other organizational constructs listed below. The sample was used to perform CFA for the WAFM in terms of individual-level perceptions and to provide convergent and discriminant validity indices for its subscales. Descriptive statistics of this sample are reported in Table 3. It is worth noting that the age distribution of the respondents represents a relatively high proportion of older workers, as 37.2% of the respondents were aged 55 and older (*M* = 50.4, *SD* = 10.6), compared with 14.5% in the total Israeli labor market (Israel Central Bureau of Statistics, 2017).

**Table 3.** Descriptive Statistics of the Main Sample in Study 3

Organization		1	2	3	4	5	
Industry		Chemicals	Media	Agriculture	Nursing services	Finance	Total sample
% of employees aged 55+		29.9%	23.0%	23.0%	32.0%	19.0%	26.6%
*N*		160	115	90	46	37	448
Age	Mean	51.4	49.9	50.0	49.9	49.7	50.4
	*SD*	10.4	12.0	10.5	10.4	6.2	10.6
	% 55+	46.5%	33.3%	34.4%	37.0%	16.2%	37.2%
Gender	% Women	38.1%	59.1%	46.7%	87.0%	78.4%	53.6%
Position	% Managerial	58.1%	25.2%	51.1%	60.9%	29.7%	46.2%
*Dimensions of WAFM*							
Core culture	Mean	5.46	5.14	5.39	5.85	5.09	5.37
	*SD*	1.33	1.50	1.18	1.19	1.36	1.35
Development	Mean	5.07	4.65	4.87	5.45	5.29	4.98
	*SD*	1.48	1.53	1.33	1.24	1.22	1.44
Wellness	Mean	4.96	4.12	4.31	4.77	5.01	4.60
	*SD*	1.41	1.61	1.43	1.47	1.35	1.51
Flexibility	Mean	3.30	4.31	3.36	5.01	4.25	3.84
	*SD*	1.77	1.68	1.52	1.51	1.51	1.75

*Notes:* Responses ranged from 1 (*absolutely disagree*) to 7 (*strongly agree*). WAFM = Workplace Age-Friendliness Measure.

The sample from Study 2 was also used for the validation process. As noted below, this sample included additional self-perceptions on organizational constructs related yet distinct from workplace age-friendliness, which were used to provide complementary indices of convergent and discriminant validity. The use of two separate samples allowed us to keep surveys as short as possible, as required (Hinkin, 2005).

#### Procedure

To reach the main sample, we approached the CEOs and/or the chairmen of the boards of companies listed in the Israeli Dun & Bradstreet database, with a request to send an anonymous survey to their employees via the organizational email system. Before determining whether an organization is entitled to participate in our study, we have ensured that the organizational age structure of each organization would include employees aged 55 and older at a reasonable rate of at least 15%. Two high-tech companies did not meet this threshold and thus were not included in the sample.

We provided each organization with a separate link to an online questionnaire using Qualtrics secure survey software, so that the responses were stored on an external server, thus minimizing biases. All English-version questionnaires were cross-translated into Hebrew (International Test Commission, 2017). All employees were asked to participate in the survey on a voluntary basis. They were told that their organization decided to participate in research about the organizational approach to work at an older age. Guidelines for participants were the same as in Study 2. The response rate was 19.9%, falling within acceptable ranges (Baruch & Holtom, 2008). The anonymity of responses was maintained on a personal level. The organizational level was kept confidential.

An acceptable three-phase approach was used to demonstrate construct validity: (a) verifying dimensionality fit and internal consistency, (b) assessing convergent validity, by denoting relatively strong correlations with other measures of related constructs, and (c) assessing discriminant validity, by denoting relatively weak correlations with measures of unrelated constructs (Campbell & Fiske, 1959; Hinkin, 2005). Each of these phases is discussed as follows.

#### Phase 1: Dimensionality

To cross-validate the four-factor structure obtained in Study 2, we conducted a CFA using SPSS Amos 21.0 on the main sample. We used centered variables to basically take into account the organizational level, so that responses in each organization became relative to the organizational means, thus easier to interpret at the individual level (Enders & Tofighi, 2007). Following the accepted recommendation to interpret multiple fit statistics (Bollen, 1989; Kline, 2010), we examined the chi-square test and the (standardized) root mean square residual (SRMR), along with the root mean square error of approximation (RMSEA; Browne & Cudeck, 1993), the comparative fit index (CFI; Bentler, 1990), and the normal fit index (NFI; Bentler & Bonnet, 1980).

##### Results.

—The initial CFA showed a moderately good fit, but with a slight need for model improvement (χ ^2^ = 929.53, *df* = 246, *p* < .00; SRMR = .04; RMSEA = .08; CFI = .92; NFI = .89). The modification indices of this model did indeed reveal that an estimation of the standardized residual covariances between sampling errors of three pairs of items representing tangential issues (8–9, 10–13, and 17–18) can yield a better fit. Reactivating the model with these estimations led all fit indices to fall within the accepted ranges (χ ^2^ = 708.50, *df* = 243, *p* < .00; SRMR = .04; RMSEA = .06; CFI = .95; NFI = .92; Kline, 2010). An alternative five-factor model resulted in a less favorable fit (χ ^2^ = 953.49, *df* = 240, *p* < .00; SRMR = .06; RMSEA = .08; CFI = .92; NFI = .89). In the adjusted four-factor model, the average correlations between the latent constructs and the observed items ranged from .77 (for core culture and wellness) to .80 (for development) and .84 (for flexibility) and supported the above fit indices. We also observed strong correlations between the latent constructs, ranging between .54 and .87, with an average of .71. All four dimensions had acceptable internal consistency reliabilities ranging between .88 and .93 (Table 4).

**Table 4.** Zero-Order Correlations and Cronbach’s Alphas in Study 3

Variable	Core culture	Development	Wellness	Flexibility	α
Age	.01	.02	.05	.03	
Gender (0 = Male, 1 = Female)	.08	.11*	.03	.09	
Position (0 = Nonmanagerial, 1 = Managerial)	−.09*	−.11*	.03	.08	
Core culture		.72**	.68**	.47**	.93
Development			.75**	.45**	.92
Wellness				.59**	.90
Flexibility					.88
Negative POS	−.53**	−.52**	−.41**	−.30**	.90
Positive POS	.60**	.56**	.66**	.43**	.91
CFD	.55**	.55**	.53**	.36**	.84
Workplace age stereotypes	−.53**	−.55**	−.46**	−.34**	.84
Quantity of intergroup contact	.43**	.34**	.26**	.10*	.79
Quality of intergroup contact	.41**	.33**	.27**	.11*	.89

*Notes: N* = 448 for all scales except POS and CFD (*N* = 122). CFD = climate for diversity; POS = perceived organizational support.

*p < .05, **p < .01.

These results suggest that all four dimensions of workplace age-friendliness are distinct but related. Therefore, at this point, we moved to use the average of items for each dimension, so that each dimension is given the same weight. The final scale items are provided in the Supplementary Appendix.

#### Phase 2: Convergent and discriminant validity assessment

The purpose of this phase of the study was to proceed to the validation process of showing evidence of convergent and discriminant validity. Convergent validity refers to the extent to which a measure is related to other similar measures of a construct, and discriminant validity represents the extent to which a measure is distinct from other unrelated constructs (Campbell & Fiske, 1959; Hinkin, 2005). Appropriately, we chose four well-established constructs with which the WAFM dimensions would be likely to have differential relationships: perceived organizational support (POS; Eisenberger et al., 1986; Rhoades & Eisenberger, 2002), climate for diversity (CFD; Gelfand et al., 2005), workplace age stereotypes (WAS; Hassell & Perrewe, 1995), and intergroup contact (Allport, 1954; Voci & Hewstone, 2003). We briefly detail below the scales of each of these constructs, followed by their expected and observed relationships with the WAFM dimensions.

POS is defined as “employees’ general belief that their work organization values their contribution and cares about their well-being” (Rhoades & Eisenberger, 2002, p. 698). To measure POS, we used a short version of the survey of POS developed by Eisenberger et al. (1986), which Hofstetter and Cohen (2014) used in a Hebrew version (α = .82). This scale has seven positively formulated items (sample item: “The organization strongly considers my goals and values”) and five reverse-scored items (sample item: “The organization shows very little concern for me”). As indicated by Rhoades and Eisenberger (2002), POS is supported by antecedents such as fairness, procedural justice, and job conditions and drives consequences such as organizational commitment and desire to remain. Therefore, we would expect scores on the WAFM dimensions to be positively correlated with scores on the positively formulated POS scale and negatively correlated with scores on the negatively formulated POS scale.

CFD is defined as “employees’ shared perceptions of the policies, practices, and procedures that implicitly and explicitly communicate the extent to which fostering and maintaining diversity and eliminating discrimination is a priority in the organization” (Gelfand et al., 2005, p. 105). We used the four-item diversity climate scale developed by Pugh et al. (2008) to measure CFD (Sample item: “Managers demonstrate through their actions that they want to hire and retain a diverse workforce”; α = .76). Based on relevant evidence (Mor Barak et al., 2016), we reasonably assume a relationship between workplace age-friendliness and the openness of the organization to individuals’ diverse backgrounds in terms of gender, ethnicity, religion, culture, and so forth. Therefore, we would expect scores on this scale to be positively correlated with scores on the WAFM dimensions.

WAS are beliefs and expectations about workers based on their age, commonly referred to as a subconcept under the general umbrella of ageism (Harris et al., 2017). To measure WAS from an organizational perspective, we implemented a nine-item version of the WAS scale developed by Hassell and Perrewe (1995), which was used in a Hebrew version by Hofstetter and Cohen (2014; sample item: “In my organization there is a belief that older workers are not interested in learning new skills”; α = .86). Based on an accumulated body of knowledge of the adverse consequences of workplace ageism (Harris et al., 2017), we speculate that the presence of negative age stereotypes in the organization may undermine the implementation of an age-friendly culture. Therefore, we would expect scores on WAS to be negatively associated with scores on all WAFM dimensions.

Intergroup contact refers to the quantity and quality of face-to-face interactions between members of clearly defined minority and majority groups (Allport, 1954; Pettigrew, 1998). We adapted an acceptable instrument from Voci and Hewstone (2003) to measure Inter-Age Contact (IAC) with older workers. The instrument comprises two four-item scales, for quantity and quality of contact (sample items: “In everyday work, how often do you interact with older workers? (never – very often)”; “How do you feel about the contact you have at work with older workers? (involuntary – voluntary)”; α = .82). Emerging evidence suggests that under certain conditions (such as egalitarian cooperation situations, pursuit of common goals, and organizational support), IAC in work settings may contribute to reducing prejudices toward older workers, which may provide fertile ground for the adoption of an age-friendly culture (Boehm et al., 2014; Kunze et al., 2011). Accordingly, we would expect scores on the IAC scales to be positively associated with scores on the WAFM scales in a manner similar to CFD, but to a lesser strength.

##### Results.

—Table 4 reports internal consistency reliabilities and zero-order correlations of the study variables. As this table reveals, the relationships between the WAFM dimensions and the above four scales are largely consistent with our expectations: (a) scores on the WAFM dimensions are strongly to moderately correlated with scores on the POS scales, with mean correlations of −.44 and .56 for the negatively and positively formulated POS scales, respectively; (b) scores on the CFD scale are strongly to moderately correlated with scores on the WAFM dimensions, with a mean correlation of .50; (c) scores on the WAS scale are strongly to moderately correlated with scores on the WAFM dimensions, with a mean correlation of −.47; and (d) IAC scores are moderately correlated with scores on the core culture, development, and wellness scales, with mean correlations of .34 for quantity and quality of contact. However, contrary to our expectations, IAC scores are weakly correlated with scores on the flexibility scale (*r* = .10, *p* < .05 for quantity of IAC; *r* = .11, *p* < .05 for quality of IAC).

## Discussion

In light of the increasing proportion of older workers in the workforce of a variety of developed countries, the purpose of the three multiphased studies described previously was to develop and validate a measure of workplace-based age-friendliness from an organizational perspective. In this article, we have provided substantial evidence to accomplish this purpose. We drew on a culture-driven model of the AFW, stemming from the integration of theoretical and empirical knowledge. A four-dimensional construct of workplace age-friendliness resulted from the first two stages of the research and thus largely supported our theoretical model. Furthermore, CFA showed that sampled organizational data fit this dimensionality in an acceptable manner.

In addition, the pattern of findings in Study 3 provides preliminary evidence of convergent and discriminant validity, along with three noteworthy insights. First, the WAFM possesses strong interdimensional correlations (Table 4). Second, nearly all the constructs from Phase 2 of Study 3 are more closely correlated with the core culture dimension than with the climate-related constructs, especially when compared with the flexibility dimension. Together with generally higher scores on the core culture scale than on all climate-related scales (Table 3), these findings correspond to the process theory of culture and climate (Ostroff et al., 2013) used to construct the AFW model. Finally, the WAFM dimensions are insignificantly or poorly correlated with demographic characteristics of age, gender, and position, indicating unbiased perceptions of workplace age-friendliness among respondents regarding these characteristics.

It should be noted, however, that the primary dimensions of the AFW model have undergone a few revisions based on empirical results from the reported studies. First, the original model’s sustainment and modification dimensions were omitted from the resulting construct and replaced by wellness. The wellness dimension justifies its label by including practices such as workplace health and well-being promotion, job redesign, and job efficacy, which were originally associated with sustainment and modification dimensions. Second, in relation to the upper part of the model’s typology, no distinction was found between sustainment practices of updating skills and knowledge and various aspects of development. Finally, in the flexibility dimension attributed to indirect adaptation to the reduced functionality of older workers, only three items survived. Although the latter outcome allows us to achieve adequate internal consistency reliability, it somewhat limits the testing of homogeneity of items (Hinkin, 2005). This pattern of results requires a reconsideration of certain forms of our organizing model, together with further validation of WAFM’s revised dimensionality in future studies. Further research is also required to revalidate our new measure over time, in different labor force settings.

The development of the WAFM introduced in this study has a number of notable implications. Our main proposed direction lies in using this measure to assess workplace age-friendliness in different organizations, in research as well as in practice, as part of efforts to maintain organizational effectiveness, and reinforce a desirable culture. As such, considerable added value can be brought by using the WAFM among a broad sample of organizations, which will allow an implementation of a multilevel approach to data analysis. To date, studies that have investigated organizational alignment with the aging and older workforce have not explored this direction (Boehm et al., 2014; Kooij et al., 2014), which is necessary for a better understanding of workplace age-friendliness, its antecedents, and consequences.

We therefore view this study as only a first step in preparing the ground for diagnosing the culture of organizations so as to accommodate the constructive inclusion of older workers. Alongside this process, the WAFM can be adopted as part of an involvement of policymakers at the organizational and/or national level who are charged with promoting employment equality for older persons. A possible implication could be the application of this measure by organizations to better understand where their strengths and weaknesses are in relation to the various dimensions and aspects associated with the beneficial employment of an aging and older workforce. Moreover, using the measure continuously may provide organizations with an assessment of the progress of efforts to improve and maintain workplace age-friendliness, thereby receiving feedback for further research and practice.

Despite the potential implications described, several limitations of measuring workplace age-friendliness should be noted. One noteworthy limitation is that workplace age-friendliness can be measured only in organizations that employ a reasonable percentage of older workers, compared with the share of older workers in the labor market in relevant occupations. That is, the presence of older workers in organizations is a necessary condition for measuring workplace age-friendliness as defined in this study. Supplementary age-related measurements can be developed for other organizations with a younger workforce structure, addressing concerns such as reduced job mobility opportunities, and organizational entry barriers for older workers (van Dalen et al., 2015).

A further limitation in this context may rest on the fact that the main sample of Study 3 involved large and leading organizations with a relatively high percentage of older workers, where the topic of work at an older age fell on attentive ears. Although it is worth learning from those organizations, a certain bias may have been included in the perceptions of employees of the sampled organizations. Future research should address this issue and include more age-diversified organizations.

A final related limitation of this study stems from our opening definition of workplace age-friendliness, which focuses on adjustments for aging and older workers rather than for workers of any age. This approach is theoretically and empirically based on unique problems and needs common to older workers and distinct from those of younger workers (Kite et al., 2005). We believe that a different approach that includes all ages would have neutralized the capture of the specific problems in the employment of an aging and older workforce. However, an alternative age-oriented approach may be useful in addressing whether employees of all ages in the organization are equally valued. In summary, we believe that the measure developed in this study has a significant contribution to researchers in the emerging field at the intersection of aging and work.

## Supplementary Material

igaa024_suppl_Supplementary_Material
