# Supplementary material for: Development and Validation of a Workplace Age-Friendliness Measure
Source: Innov Aging. 2020 Jul 1;4(4):igaa024. doi: 10.1093/geroni/igaa024 (PMC7413617; doi:10.1093/geroni/igaa024)
Supplement: igaa024_suppl_Supplementary_Material [file igaa024_suppl_supplementary_material.pdf]

Online Supplementary Material

Appendix

Final Workplace Age-Friendliness Scale Items

**Measure**
<u>Age-Friendly Core Culture</u>
My organization treats older workers fairly and equally.
In my organization there is no age discrimination in processes such as recruitment, promotion, and dismissal.
Managers in my organization are a personal example of the wish to recruit and retain workers of all ages, including older workers.
In my organization, there is a positive atmosphere towards the employment of older workers.
My organization promotes multi-age diversity in the organizational workforce.
My organization makes sure that older workers are recognized and respected no less than other workers.
My organization shows responsibility for older workers who have long contributed to the organization.
Older workers in my organization are not the first priority for dismissal during organizational change or downsizing.
Older workers in my organization are not pressured to vacate their place and retire early.
<u>Development</u>
My organization allows older workers to update and upgrade their knowledge and skills as part of their job.
In my organization, older workers are encouraged to acquire more new skills appropriate for changes in their professional field.
In my organization older workers are encouraged to serve as mentors for other employees.
My organization allows older workers to continue to develop throughout their working lives.
In my organization, older workers are encouraged to initiate changes in their jobs, in line with the needs of the organization.
My organization knows how to benefit from the total knowledge, skills and abilities of older workers.
<u>Wellness</u>
My organization takes care and acts to promote the health and well-being of older workers.
My organization encourages older workers to participate in health promotion activities.
My organization works to raise awareness and change attitudes towards continuing work at older ages.
In my organization, older workers are offered job changes, if necessary, to better fit their abilities.
My organization organizes the work so that older workers remain in the organization in optimal functioning.
When required, my organization helps to reduce or adapt physical or psychological efforts to the abilities and needs of older workers.
<u>Flexibility</u>
In my workplace, older workers are given flexibility in choosing the range of hours worked.
In my workplace, older workers are given flexibility in choosing the scope of the position.
In my workplace, older workers are given flexibility in choosing the job location.

Note. The original Hebrew-version questionnaire was cross-translated into English (International Test Commission, 2017).

Source: Raphael Eppler-Hattab, MSc, Israel Doron, PhD, Ilan Meshoulam, DBA, Development and Validation of a Workplace Age-Friendliness Measure, *Innovation in Aging*, Volume 4, Issue 4, 2020, igaa024, https://doi.org/10.1093/geroni/igaa024.
